# “My Life in the Hospital”: Narratives of Children With a Medical Condition

**DOI:** 10.5334/cie.12

**Published:** 2021-02-03

**Authors:** Michele Capurso, Federico Bianchi di Castelbianco, Magda Di Renzo

**Affiliations:** 1 Università degli Studi di Perugia, IT; 2 Istituto di Ortofologia, IT

**Keywords:** Children’ narrative, hospital, play, relationships, interpretative phenomenological analysis

## Abstract

Pediatric hospitalization is a common experience that may increase children’s sense of isolation and impinge on their social-emotional wellbeing. Educators and medical practitioners could minimize these negative effects of hospitalization if they were able to listen to the voices of the children and, therefore, better meet their needs. This qualitative study provides an overview of how children with a medical condition actively construct and organize their thoughts and feelings about illness, life in hospital, and relationships. We extrapolated from a collection of children’s narratives from a previous more comprehensive study (consisting of 379 narratives from children in 29 public hospitals across Italy, age range 3–14 years). Narratives grouped under the headings “Me and my illness” or “Me and the others” were selected and analyzed using interpretative phenomenological analysis (IPA) to identify the richness and complexity of children’s experience. Results showed that children’s description of their illness was affected both by cognitive and social factors. For children, the concept of feeling ill or well is not linked only to the fact that they are in hospital for a medical condition; rather, it is influenced by their ability to form relationships with others, play, be active, and feel alive within the hospital environment. Listening to narratives can deepen our understanding of children’s illness-related experiences and how they make sense of their situation. A set of practice implications are presented to help health professionals and educators to improve their listening capabilities and better prevent adverse pediatric hospitalization outcomes.

*When a child enters a hospital, it is as if he were dragged into a forest, far away from home. Some children remember to fill their pocket with pebbles and drop them along their path to create a trail so that they can find their way back home even under the moonlight. However, some children do not know fairy tales and cannot make good use of the pebbles. Those children leave only small bread crumbs on to mark their path. It is a fragile trail that even ants can easily erase. These children get lost in the woods and cannot find their way back home anymore*. Adapted from (Canevaro, 1976, p. 27)

Hospitalization is a common experience among children. According to the data from the U.S. Healthcare Cost and Utilization Project, in 2012, approximately 1.8 million children experienced hospitalization, with an average length of stay of 4.2 days (Witt, Weiss, & Elixhauser, 2014). Considering that the number of children in the United States at that time was 73.7 million (Child Stats, 2017), we estimate an incidence of pediatric hospitalization of 2.4% per year. Additionally, 10% to 20% of children are estimated to suffer from a chronic medical condition, a situation that often leads to repeated hospital admissions (Compas, Jaser, Dunn, & Rodriguez, 2012; Ward, DeSantis, Robbins, Kohler, & Jemal, 2014; West, Denzer, Wildman, & Anhalt, 2013).

Studies have shown that the experience of illness and the related pediatric hospitalization may affect the psychosocial wellbeing of children and families (Barlow & Ellard, 2006; Obaid, 2015). Specifically, hospitalization may increase children’s sense of isolation and may impinge on their independence, their usual self-care abilities, and their self-control (Coyne, 2006). Further, some of these effects may remain after a child has been discharged (Martínez-Mejías, Úriz, Rivera-Pérez, & Garolera, 2017). These far-reaching consequences call for professionals to take responsibility for planning developmental instigative interventions that can foster children’s resiliency and sustain their healthy psychosocial development (Barakat, Pulgaron, & Daniel, 2009; Roberts, Aylward, & Wu, 2014).

Such interventions could be built if practitioners were aware of, and took into consideration, children’s perspectives when in a healthcare setting. Indeed, international legislation on children’s rights asserts the need to involve children in their own healthcare-related processes (Lansdown, 2011; Lansdown, Jimerson, & Shahroozi, 2014). Thus, Article 12 of the United Nations Convention on the Rights of the Child (1989) states that children have a right to express their views and have them taken seriously, in accordance with their age and maturity. Consequently, several scholars have proposed that healthcare services and policies be centered on the needs of the children and their families, and that children be involved when decisions are made that concern their own care (Boztepe, Çınar, & Ay, 2017; Lansdown, 2011; Quaye, Coyne, Söderbäck, & Hallström, 2019). While the legal and psychosocial basis for listening to children is well established, studies that take proper account of the patient’s perspective in pediatrics are relatively rare. Besides, the voice that surfaces in existing studies is often channeled through a proxy (nurses or the parents), and is rarely the one used by the children themselves (Boztepe et al., 2017; Quaye et al., 2019).

However, listening to children and presenting their own voices is important, as research has shown that individuals who have the opportunity to tell stories that feature themes of personal agency – active role-taking in meaningful activates – tend to demonstrate higher levels of positive mental health, wellbeing, and maturity (Capurso, 2015; McAdams & McLean, 2013). Listening and understanding the essence of children’s hospital-related experiences is a critical aspect with regard to several areas of their healthcare and educational development.

In terms of clinical care, there is a recognized need to identify strategies that will support children’s involvement in tailoring their healthcare services, and this can only be achieved by listening to their voice (Quaye et al., 2019). As noted by Coyne (2006), such efforts will ultimately improve the quality of care provided for children and, consequently, their entire family. From a developmental point of view, if we want children to become adults who actively manage their health, we need to give them a sense of agency from a young age (Goldhagen, 2003). This starts with listening to them, and showing them that their opinions matter and that they can impact reality by sharing their thoughts (Lansdown, 2011). Finally, from an educational point of view, it has been established that children who learn to speak and think about their own experiences become active and reflective citizens later on in life, because they gradually learn that they can influence their own environment (Jans, 2004).

## METHODOLOGICAL ISSUES IN LISTENING TO CHILDREN

Listening to children is not a straightforward process, because it requires the integration of several components of the child’s psychology and the incorporation of different points of views (Fargas-Malet, McSherry, Larkin, & Robinson, 2010; Jadue Roa, Whitebread, & Gareca Guzmán, 2018). Qualitative research with children who talk about their hospital and illness experiences must take a set of crucial aspects into consideration. From children’s perspective, one needs to know their way of understanding their illness and how it is connected to their way of making sense of reality. From the researcher’s perspective, there is the challenge of helping children tell their reality and make sense of it, using language and a communication mode that is compatible with their developmental stages and psychology. Then, once narratives have been collected, there is the issue of describing and interpreting children’s accounts of their experiences in relation to their context.

This twofold problem, involving both the children’s and the adults’ perspectives, may be described as double hermeneutics, which is a central aspect of many interpretative research methods (Smith, Flowers, & Larkin, 2009). A key aspect of interpretative research is that “the participant is trying to make sense of their personal and social world; the researcher is trying to make sense of the participant trying to make sense of their personal and social world” (Smith et al., 2009, p. 40). The fundamental methodological factors that are at play when listening to children talking about their illness are analyzed in the following paragraphs.

### CHILDREN’S UNDERSTANDING OF ILLNESS

Children can understand illness in two main ways (Capurso, 2019; Rushforth, 1999).

The first is associated with Piaget’s genetic epistemology, and connects children’s understanding of illness to their cognitive stage of development (Piaget, 1953). According to Bibace and Walsh (1980, 1981), children aged 2–6, who are in the preoperational Piagetian stage, see illness in a similar way and use a phenomenistic or magical contagion explanation of how they became ill. Children in the concrete-operational stage (age 7–10) use more specific illness-related concepts that are somehow connected to reality, and the concepts of internalization and contamination become common. Only formal operational children (age 11+) appear to understand the generalized principles of infection, health maintenance, and treatment.

More recently, the Piagetian developmental theory has been replaced by a more dynamic and sociocultural view of child development. Authors such as Vygotsky (1978), Bruner (1991), and Bronfenbrenner (2005) have identified links between the children’s emotional, social, cultural, and cognitive experiences and their ability to actively build a new understanding of their reality. These theories emphasize the child-in-context, highlighting the way in which children actively construct their thoughts through social and cultural experiences. This, in turn, has informed a new view of the continuously evolving capabilities of children. According to this view, which we term the *social-constructivist* view, children have the potential to grasp and understand complex concepts if they are connected to their context in the right way and if they are provided with cultural and cognitive mediators that help them connect this reality to their prior knowledge (Dudek, Strobel, & Runco, 1993; Walberg, 1969). This view has been used to explore new possibilities in children’s understanding of illness that differ from the supposed cognitive limitation of age (Capurso, Lo Bianco, Cortis, & Rossetti, 2016; Leonhardt, Margraf-Stiksrud, Badners, Szerencsi, & Maier, 2014; McIntosh, Stephens, & Lyons, 2012, 2013; Siegal & Peterson, 2005).

### MAKING SENSE OF REALITY

#### Helping children tell their illness-related reality

Several aspects need to be considered to help and support children in telling their own version of their reality.

The first is connected to language and the ability of children to express themselves. While it is accepted that children experience the same array and range of feelings as adults (Harris 1989), it is also established that they experience difficulty when asked to verbalize the reason for their discomfort and to describe their emotions (Harris, Olthof, & Terwogt, 1981). Young people’s worries are frequently amplified by the inability of adults to recognize children’s emotional experiences at a deeper level and, therefore, address their need for protection (Dolto, 1985). This inability often results in adults considering their own view of the world as a proxy for the child’s perspective (Capurso, 2019; Sartain, Clarke, & Heyman, 2000).

The communication gap also increases the lack of understanding. For example, adults expect children to express themselves via the spoken language, using a logical and linear form of thought. However, this expectation takes for granted the ability to use and master a more abstract form of thought; however, the reality is that children are only able to master this as they age and become more mature (Lemerise & Harper, 2014). A key instrument that humans use to make sense of reality is narrative thought. Bruner (1986) argued that creating a narrative is not a passive action; on the contrary, he claimed that through narratives, humans actively construct their own worlds. It is through the telling of stories that humans, ultimately, come to answer the fundamental question, “How do we endow experience with meaning?” (Bruner, 1986, p. 12). In children, the ability to express themselves increases when they have the freedom to communicate through an array of methods, such as storytelling, drawing, play, and talking about their direct experiences (Angell, Alexander, & Hunt, 2014; Capurso & Ragni, 2016; Einarsdottir, Dockett, & Perry, 2009).

#### Interpreting how children make sense of their experiences

On the part of the researcher, the need to gain an understanding of the situation from the child’s unique frame of reference may be satisfied through the use of interpretative phenomenological analysis (IPA; Smith et al., 2009). Intended to discover the meaning that a particular experience, event, or situation holds for a specific individual, IPA allows the researcher to reveal how participants make sense of their personal and social world (Smith & Osborne, 2015). To date, use of this methodology has been limited to children with cancer and oral interviews (Griffiths, Schweitzer, & Yates, 2011) and the present study was designed to expand the use of IPA to a more general pediatric population and to the interpretation of children’s drawings.

### THE PRESENT STUDY

The present study represents a secondary, in-depth analysis of data gathered for a previous mixed-methods study of children’s narratives about their hospital experience (Bianchi di Castelbianco, Capurso, & Di Renzo, 2017). The original study collected narratives from 379 children in 29 public hospitals across Italy (age range 3–14 years). In the original study, children were invited to create a narrative, through writing or drawing, on a subject chosen from a list presented to them (Angell et al., 2014). The list comprised several themes, including the perception of the outside world (“the world outside my hospital window”), friendship, school, and family representation (“my school,” “my friends in the hospital,” or “my family and I”), play (“my play activities in the hospital”), or different aspects relating the representation of their illness (“I explain my illness,” “I draw my illness,” “my illness is like”). During the original study, children were approached through local contacts who acted as the mediators (Grant, 1977). Mediators were free to contact the children at the appropriate time and in the best way (usually in small groups in a playroom, after obtaining parental consent), taking the child’s current situation into account and with the directive not to put undue pressure on the children. The mediator also acted as a trusted adult who was there to listen to the child’s narration without judgment. In the primary study, children’s narratives were eventually analyzed for content and organized into thematic content clusters.

The present study aimed to gain an understanding of the illness- and hospital-related experiences of children as they attempted to make sense of their present reality through narratives. Besides, the study aimed to shed light on the social and cognitive models behind children’s understanding of illness by considering how children’s accounts of their illness and hospital life were consistent with these models. Specifically, our goal was to answer the following research questions:

1. How do children depict their illness? How do they explain it to others?
2. Do children depict their illness only on a cognitive basis or do social and emotional factors play a role in their perception and the spontaneous description of their illness?
3. How are relationships with others perceived, and how do they help children cope with hospitalization?
4. What meaning does play assume in the eyes of the hospitalized child?

## METHOD

To address the research questions, the research group selected a variety of children’s work from the primary study based on the following criteria: (a) The subject of the chosen work was either “Me and my illness” or “Me and the others”; (b) Selected works represented a balanced distribution of age, gender, and cognitive development of the participants; (c) Selected works equally represented positive and negative accountings of the children’s situation; and (d) Selected works reflected the wide range of activities, strategies, and ways children use to make sense of their reality recounted in the primary research.

### ANALYSIS

Whereas the primary study (Bianchi di Castelbianco et al., 2017) gave a general overview of children’s experiences while hospitalized, the depth, richness, and texture of children’s narrative were lost within the wider thematic clusters generated by the content analysis. This led to the decision to conduct this secondary study, which would provide a more in-depth account of the children’s narratives. For this reason, the selected narratives were interpreted using IPA (Smith et al., 2009), which focused on children’s lived experiences and the meaning of having to live in the hospital for a while. Considering that IPA is not a prescriptive approach, but offers a set of adaptable guidelines that can be tailored by researchers based on their research aims (Smith & Dunworth, 2002), the present study expanded the use of IPA to include children’s definition of illness and their perceptions of their social connections while hospitalized. It also moved beyond verbal interactions by including a set of different communication media, such as drawings, storytelling, and any other methods of narrative expression the children felt comfortable using.

Considering the flexibility allowed by IPA, the analysis was carried out in three main phases that reflected the fact that this was a secondary study based on a pre-organized set of data. In the first phase, the researchers read and examined the children’s narrative artefacts repeatedly, with the aim of describing the content of the data, each time annotating the text or pictures with initial descriptive comments. During the second phase, the researchers looked at how the narratives connected with each other, and with one of the selected themes (“Me and my illness” or “Me and the others”), which had already been labeled in the previous primary research (Bianchi di Castelbianco et al., 2017). This led to the third stage of analysis, where the researchers were more interpretive, by forming questions and writing conceptual comments on how the chosen theme, the present illness, and the hospital context would connect in the analyzed narrative of the child. To better understand the children’s drawings, a phenomenological approach to the analysis of the drawings was adopted (Malchiodi, 1998). As a result, and in line with the phenomenological nature of the analysis, the final comments on the children’s narratives reported here reflect not only the participants’ original words and thoughts but also the researchers’ interpretations.

## RESULTS

Based on our research questions, this section is organized into two main areas consisting of several subsections. “Me and my illness” charts Research Questions 1 and 2; it presents narratives about the children’s perception of illness, and has been divided into the illness’ definition, an assessment of the level of understanding the illness within a Piagetian and socio-constructivist frameworks, and metaphors. “Me and the others” was developed based on Research Questions 3 and 4, and focuses on interpersonal relationships and play. In the following sections, names of children have been changed or masked to protect confidentiality.

### “ME AND MY ILLNESS”

**Figure 1:**
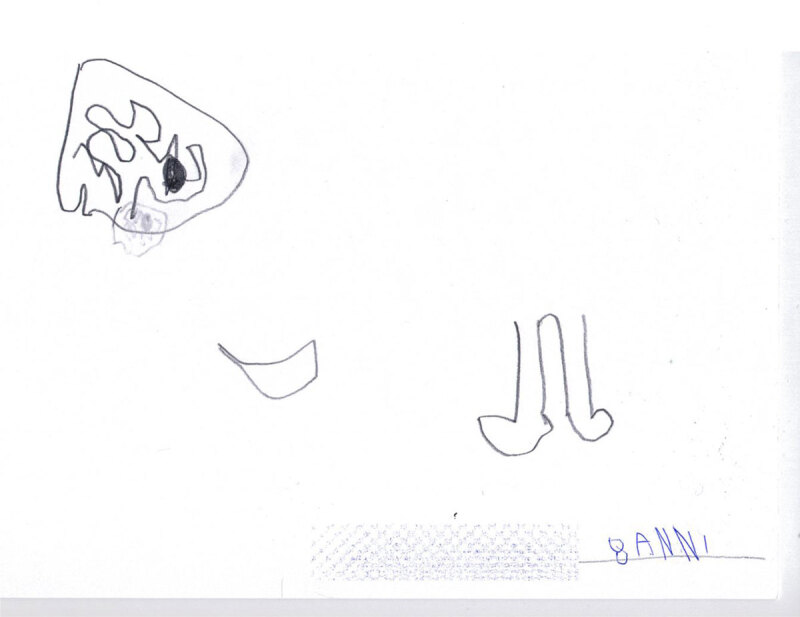
This is my brain with my little stone. This is my twisted mouth, and my bum leg. (Claudio, age 8).

Children’s drawings often reveal a great deal about their psychological state, especially when they depict themselves and their illness. For example, Figure 1 shows a very dramatic picture composed of isolated, compromised body parts (the brain, the twisted mouth, and the misshapen leg) made by an 8-year-old boy. Claudio’s image as a whole is compromised, and the illness appears as a totality without any developmental spaces. It shows a fragmented reality that permeates the very self of the child and his body image. Claudio does not perceive himself as a whole or functioning individual; he can only see his illness and broken parts. The absence of color increases the sense of depression and immobility.

When coping and psychological systems are absent, the child’s entire chronosystem perspective is affected. The dark, dysfunctional present is all that the child can perceive. There are no expectations or anticipations of a possible future because planning is not possible outside of any meaningful social or educational relationship. Such an absence of perspective (the awareness of the things I can do today and things I may be able to do tomorrow) makes the present even harder to bear. Nevertheless, our experience and the theory of resilience (Masten, 2001; Rutter, 1987) tell us that recovery remains possible if sufficient educational, environmental and psychological resources are provided.

**Figure 2:**
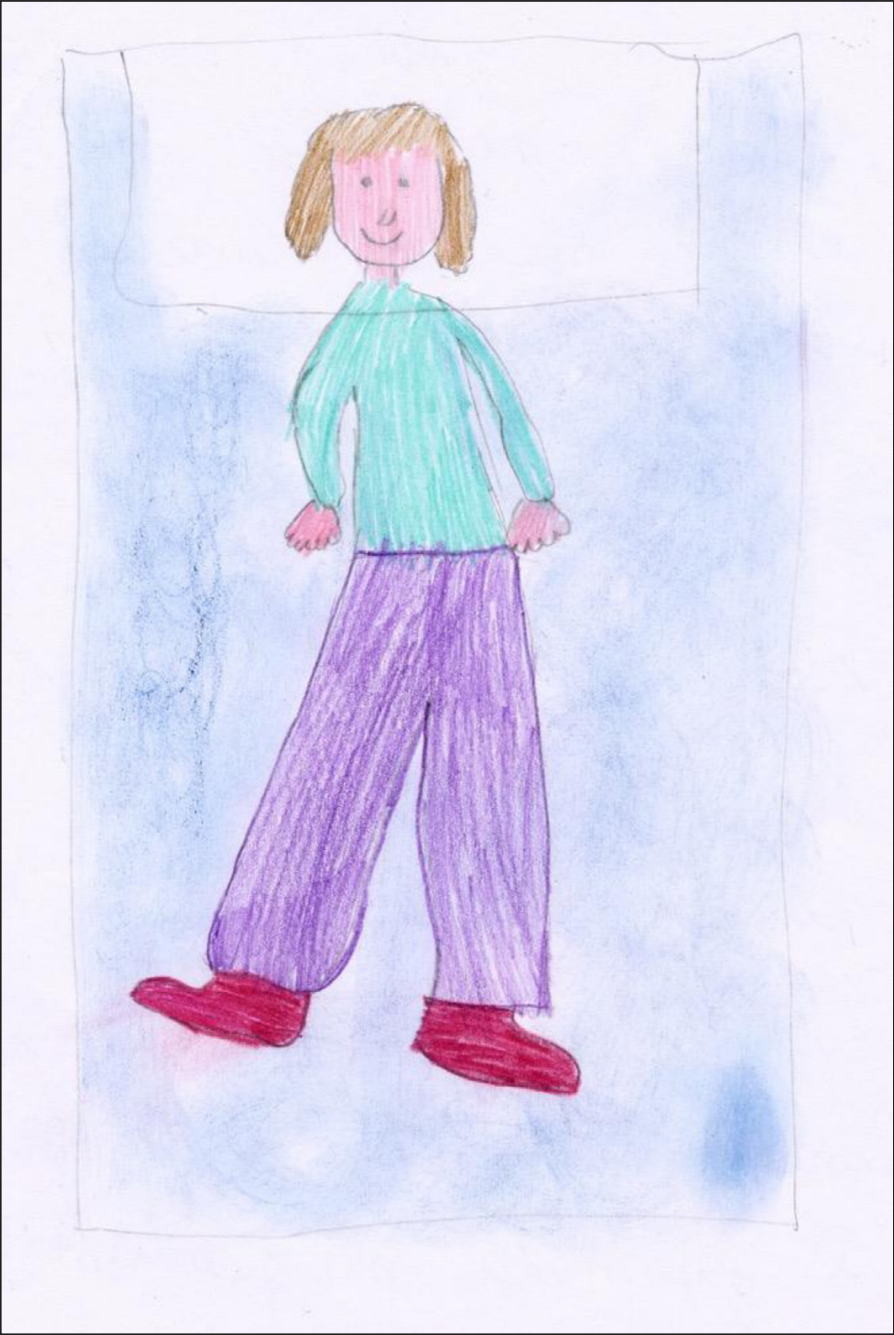
“February 20: Today is my first day in the hospital because they found out that I have diabetes, which is a sort of a disease, where nothing bad is going to happen, it’s just that I need to have some pinpricks. However, I discovered that I also have coeliac disease. February 22: Today they put me on the insulin pump, at least I don’t have to have any more pinpricks, because I only do one in my belly, and I only have to do the finger prick, so I can check my insulin and see how much I have. Tomorrow I’ll go home and on Monday I’ll go to school and see all my friends again, and we’ll play tea ladies again.” (Claudia, age 9).

In contrast to Claudio’s work is the picture drawn by Claudia, aged 9 (Figure 2). This image is full of pastel colors, with a well-shaped and proportionate body, a smiling face with some clean and tidy hair. The bed, which somehow represents the presence of the illness, is faint and is depicted in the background; it is shown as a vanishing object. Its temporary presence is destined to disappear soon, making room for everyday life, which has not stopped despite the child’s illness. Even the child’s comment is more vibrant and much more positive. Claudia explains her disease using words she has heard from her parents. Her situation is not as serious as Claudio’s, and despite experiencing some discomfort, she produces a complete narrative, full of coherent temporal references. The future is depicted as being open to opportunities and is seen as easily manageable by the child, despite her illness.

Even children with cancer are capable of finding positivity and expressing hope for the future. For example, Ivan tells us about his difficult times, having undergone three major brain surgeries, and his narrative lacks any negative perceptions. Instead, he chooses to present us with an image of a fight, in which he plays an active and vital role (the narrative is written in the first-person singular). The tale ends with the certainty of victory:

#### Children’s understanding of their illness

According to Bibace and Walsh (1980, 1981), children’s understanding of illness is linked to their level of maturity, and their narratives fall within Piaget’s developmental stages. Some of the children’s narratives in this study confirmed this theory.

For example, Francesco, age 4, has bronchitis and undergoes daily aerosol therapy. He represents his illness in an ethereal way, in line with Bibace and Walsh’s magical stage (1980; Figure 4). A genuine causal understanding of his illness is absent, and Francesco can only make sense of the illness by using a ghostly and foggy representation, similar to his aerosol experience.

**Figure 3:**
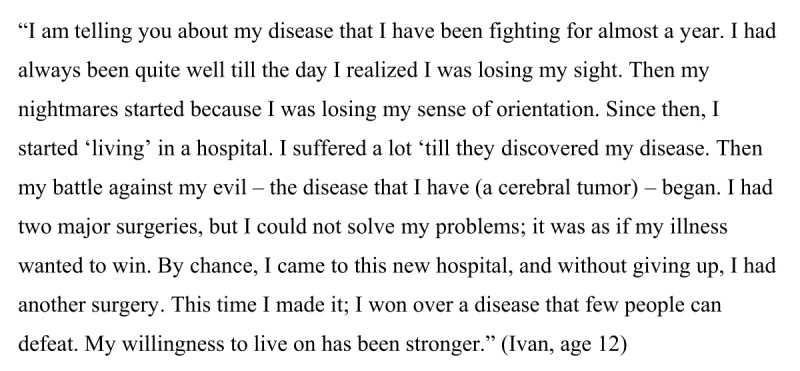
Ivan’s narrative of his experience of illness.

**Figure 4:**
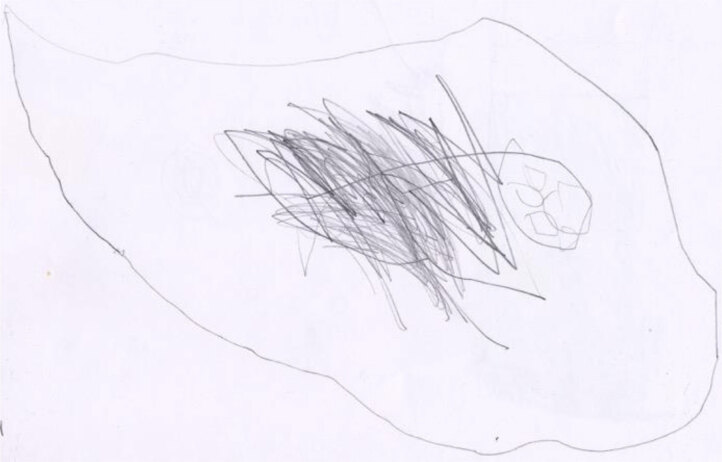
Francesco, age 4, represents his bronchitis.

**Figure 5:**
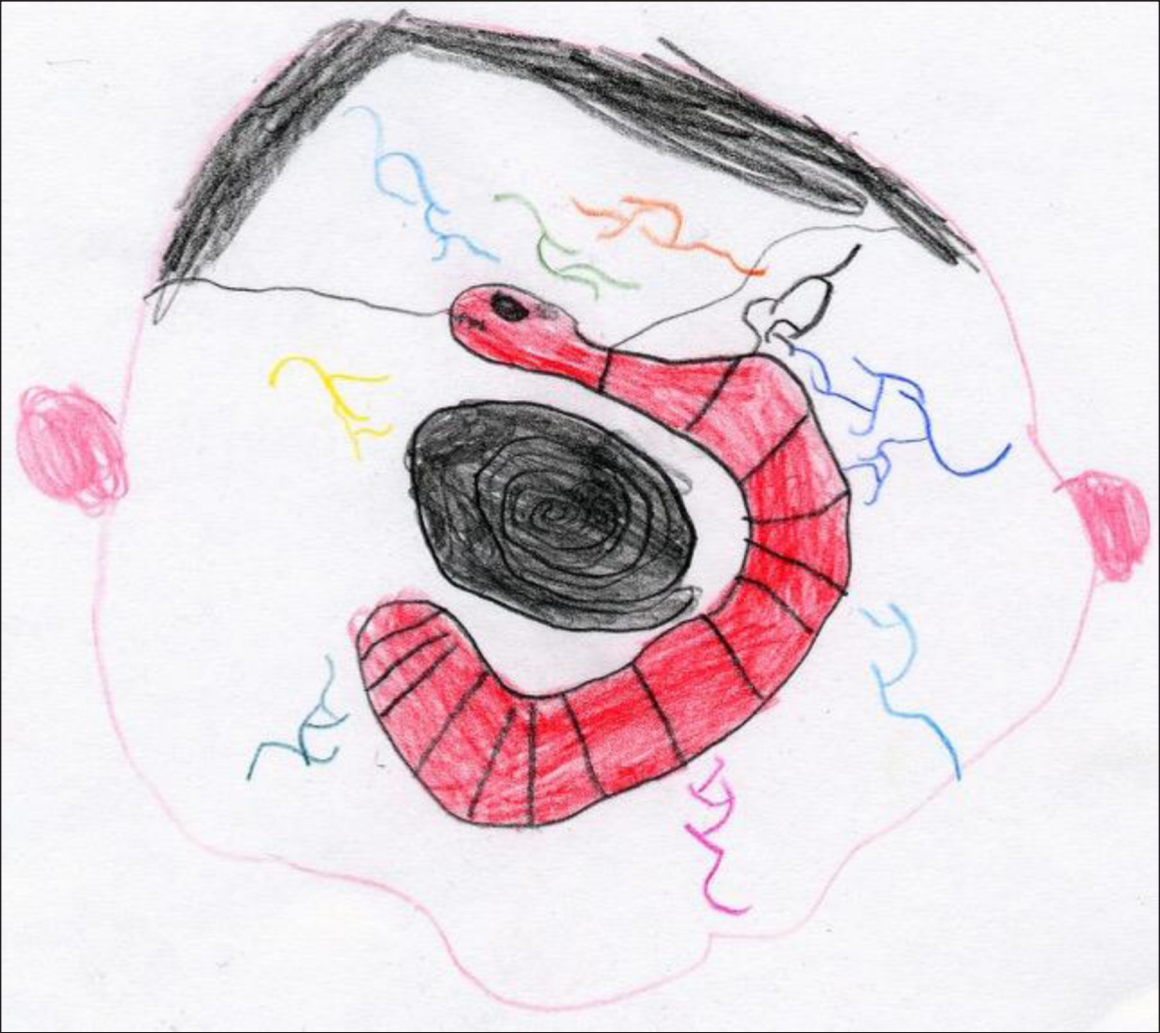
Miguel, age 6, represents his brain tumor. The child explained: “I draw my head with my ears and my hair. I also draw the brain and the veins. In there, there is the little wormy Brainiac. I think he entered here [points to his head].”

In older children, the concept of internalization becomes apparent. This can be seen in the following case of Miguel, who has a brain tumor. Miguel shows that something is inside his head, which is clearly depicted, and he also draws his ears, hair, and the veins inside (Figure 5).

**Figure 6:**
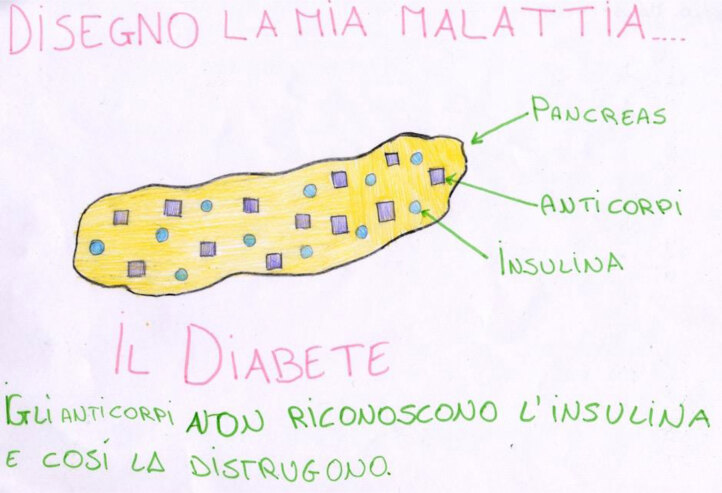
Roberta, age 11, is affected by diabetes. She depicts her pancreas, her antibodies, and insulin, and explains: “Diabetes is when antibodies fail to recognize insulin and destroy it.”

Older children are capable of giving fuller explanations of their illness because they can use more cognitive and representational tasks. For example, 11-year-old Roberta gives a very linear and clear explanation of her illness, but the connection between her pancreas and the rest of her body is missing, showing an inability to grasp a more systemic representation of the human body (Figure 6).

**Figure 7:**
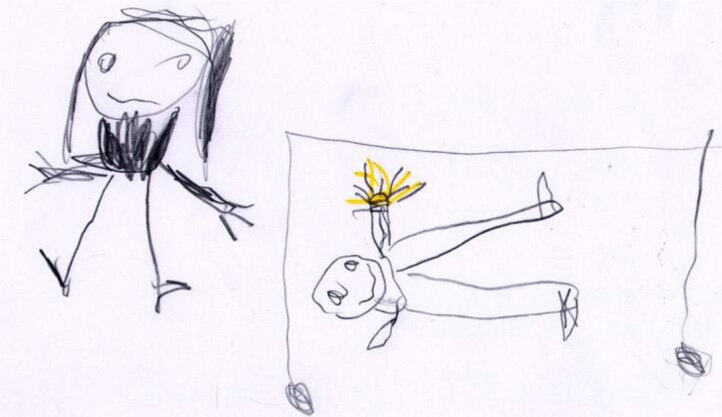
Andrea, age 5, following hand surgery. He explains to his teacher that even if his hand hurts, his mother is always there, even at night, and never leaves him.

However, not every representation identified in this study agrees with the conceptual framework of Bibace and Walsh (1980, 1981). Sometimes, in the children’s narratives, other factors exceeded the importance of the child’s age in the perception of the illness. For example, in Andrea’s representation of his illness and experience of pain (Figure 7), there is only one spot of color – the yellow outline that he chose to represent the pain in his hand. If, in chromatic terms, attention is drawn to the invasive and persistent nature of pain, in spatial terms, the scene is full; the mother and the child are both present. Despite the situation, the child is smiling (even if the smile seems a bit uncertain due to the fuzzy pencil line). The mother is also trying to smile, even though her expression is more anxious. It begs the question of whether it is the mother who is comforting the child or if it is the other way around?

**Figure 8:**
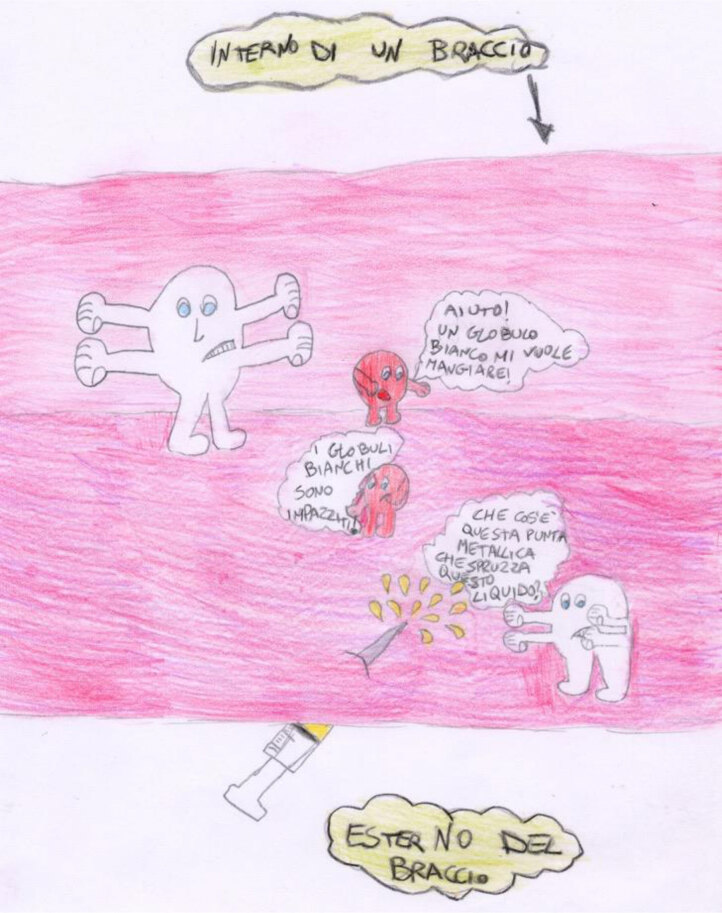
Matteo, age 11, depicts his leukemia.

Figure 8 shows the work of Matteo (aged 11, affected by leukemia). Matteo depicts his disease in a way that is coherent with his Piagetian developmental stage. Thanks to his logical-formal thinking, he can imagine the inside of his body, his white cells, his red cells, and the medicine that is starting to interact with the white cells.

**Figure 9:**
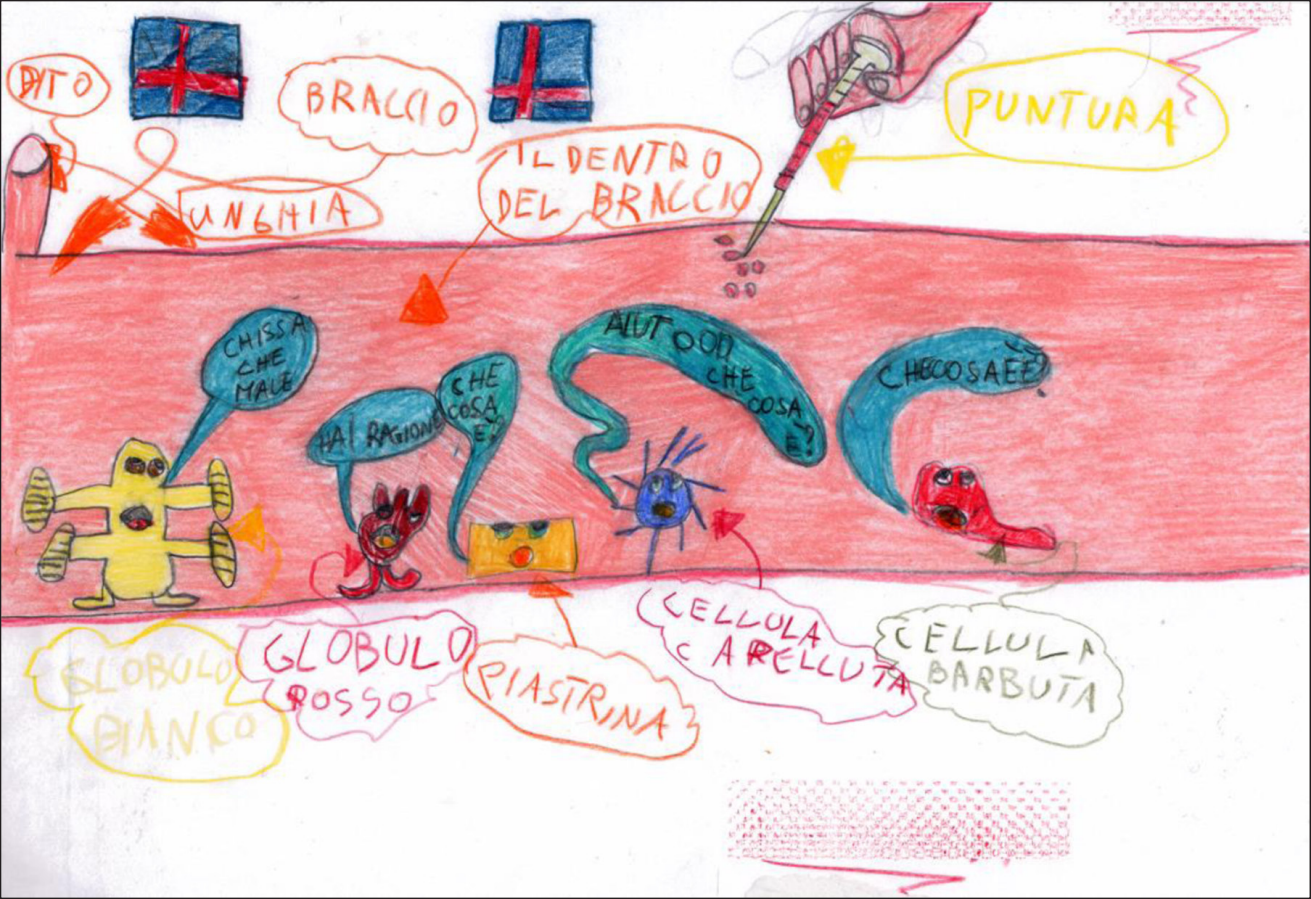
Giovanni, age 7, depicts his leukemia.

While Matteo was working on his drawing, Giovanni, age 7, came into the hospital playroom. Giovanni also chose to draw his leukemia (Figure 9). He draws a picture that is very similar to the one drawn by Matteo, but that is much richer in detail. Giovanni’s age only just places him in the concrete-operational stage and he should, therefore, not be able to comprehend and imagine the invisible parts of his body, the different cells with their different functions and the chemotherapy drug interactions with these cells, but he does. Thanks to his interaction with Matteo, he can overcome the cognitive limitations of a concrete-operational stage and work out the more complex interactions between his body, his illness, and the chemotherapy he is undergoing.

#### Metaphors for the illness

Metaphors are a compelling narrative tool, but one that is not easily grasped by younger children. Nonetheless, they provide us with powerful and vivid images. Some of the images are full of hope and open to the future; others are more dramatic.

**Figure 10:**
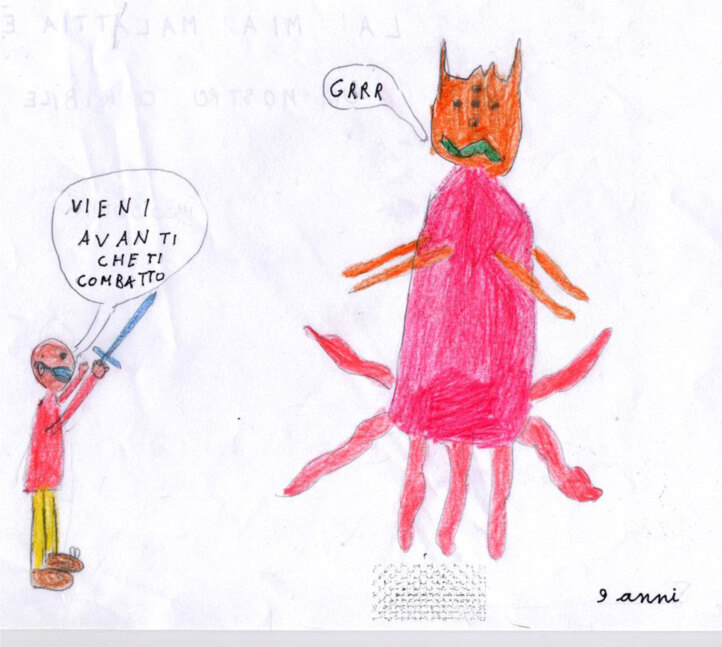
Mattia, age 9. “My disease is like a horrible monster.”

In Figure 10, Mattia, age 9, represents his illness as a terrible and overwhelming monster, depicted with vivid and belligerent colors. The monster is the main character of the picture, coming forward with tentacle arms and an evil grin. Anybody would be scared in such a situation. However, Mattia is able to overcome his fear. In a moving attempt, he does not give up; he does not run away screaming but awaits the monster with all his might. He is alone, without any hair, and a mask on his mouth, but he takes on the fight. The disproportional relationship between the monster-illness and the child is touching, but what is more poignant about this picture is perhaps Mattia’s loneliness. There is no one else around him. He has only his sword to comfort him. It is as if the child is perceiving that, ultimately, the fight is between him and his illness, that this is a venture that he is taking on alone.

**Figure 11:**
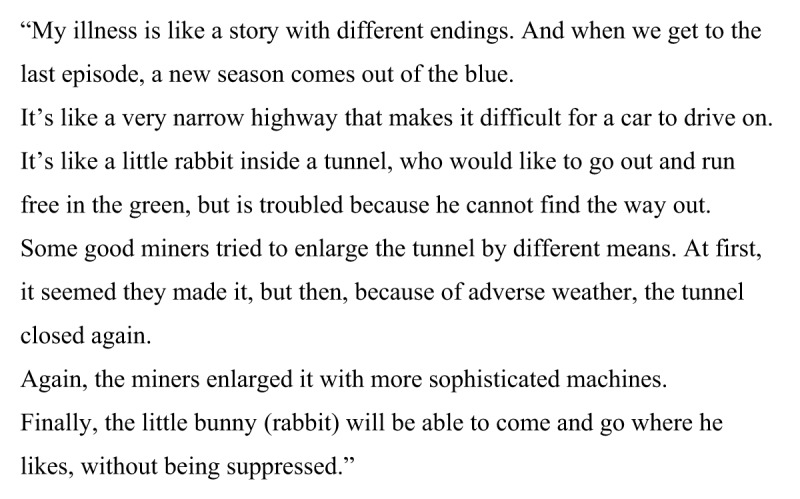
Martina, age 10. “My illness is like a little bunny rabbit.”

Martina’s metaphor (Figure 11) is entirely original, and shows a great deal of conscious thought about her situation. She knows her treatment may or may not work. She is aware that doctors have different means of curing her disease, but she also knows the disease may relapse over time. Overall, she is quite optimistic about her future because she predicts a possible positive outcome. Martina sees the illness as something vital, only trapped inside a tunnel. She does not want to attack her illness; all she wants is for the rabbit to be able to run free. As often happens with figurative language, the child subject likely oscillates between the illness and the self. The trapped rabbit could well be the child, and all she asks is to be able to run in the open as she was once able to do.

**Figure 12:**
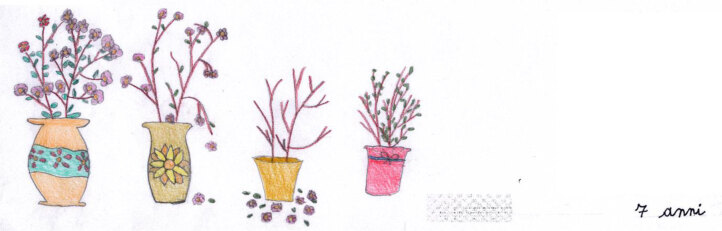
Elena, age 7. “My illness is like a flower that withers away and is then reborn.”

Elena’s metaphor (Figure 12) is linked to time and life. She sees herself as a “flower that withers away and then is reborn.” Elena’s drawing is vibrant with lots of color and hope, and the last plant on the right is sprouting, as a reminder of the life that is returning. The future remains open. The right side of the sheet is blank, but this is where she has put her signature as if she wants to reassure herself and others of her presence in the future.

**Figure 13:**
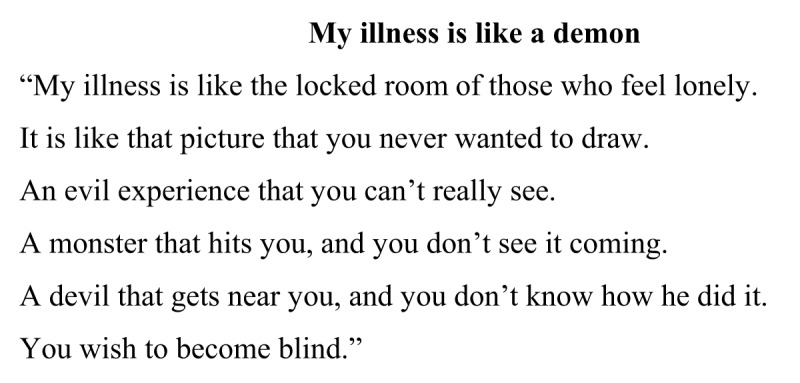
Giulio, age 10. “My illness is like a demon.”

Other children depict a much more negative view of their illness. For example, this is the case for Giulio (Figure 13), who uses a series of negative metaphors to describe his illness verbally. The experience is so negative that Giulio cannot even draw it. He only wishes to become blind, maybe to remove it from his sight (and mind) and never see it again.

### “ME AND THE OTHERS”

#### School, peers, and play make the hospital a good place

**Figure 14:**
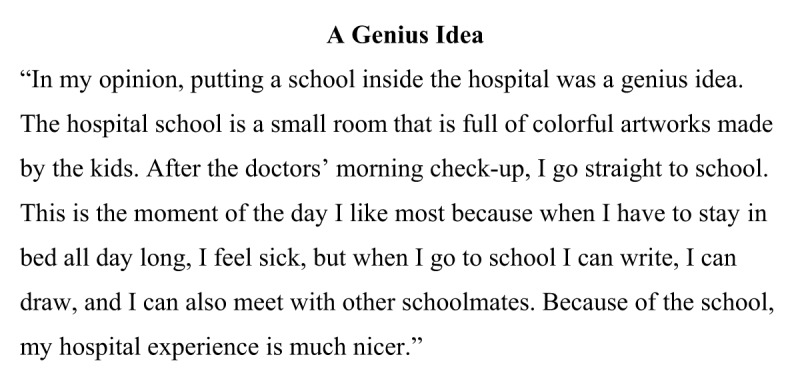
Giovanna, age 10. “School in the hospital is a genius idea!.”

**Figure 15:**
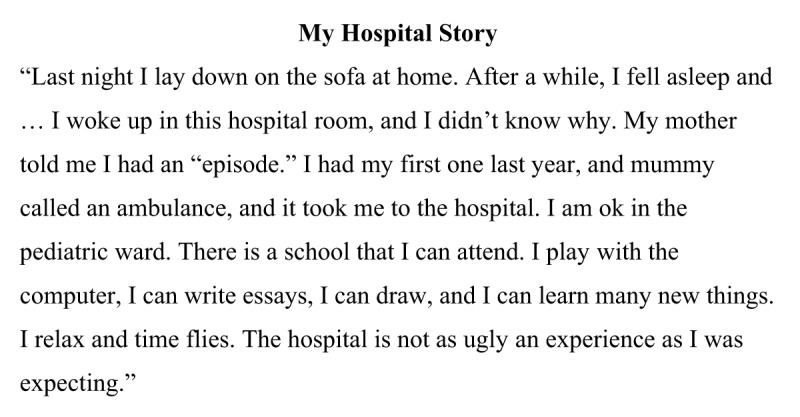
Carlo, age 9. “The hospital is not so ugly, after all.”

The presence of others is often narrated by children with elements of surprise and enthusiasm. It is not only that the company of others indicates that they are not alone in their situation, but it also means they can carry on growing, thanks to interpersonal relationships, which are the healthy parts that still exist inside them, despite the illness. Sometimes this “good part” is represented by the school, as in the essays by Giovanna and Carlo (Figures 14 and 15).

In the essay seen in Figure 14, Giovanna tells us about all the positive things she has at school, as opposed to staying in bed, which makes her feel bad. The illness experience is quickly marginalized with a short incidental sentence. The school experience takes up most of the child’s narrative. This tells us than when the environment is full of developmental opportunities, the perception of illness fades into the background.

In Figure 15, Carlo gives us a very sharp, and quick narrative of his experience with illness, but then moves on to list all the positive things he can do at school as if to reassure himself that he is still a fully functioning young boy. This short recap of all the things he can do allows the child to conclude that the hospital is not an ugly place, after all.

**Figure 16:**
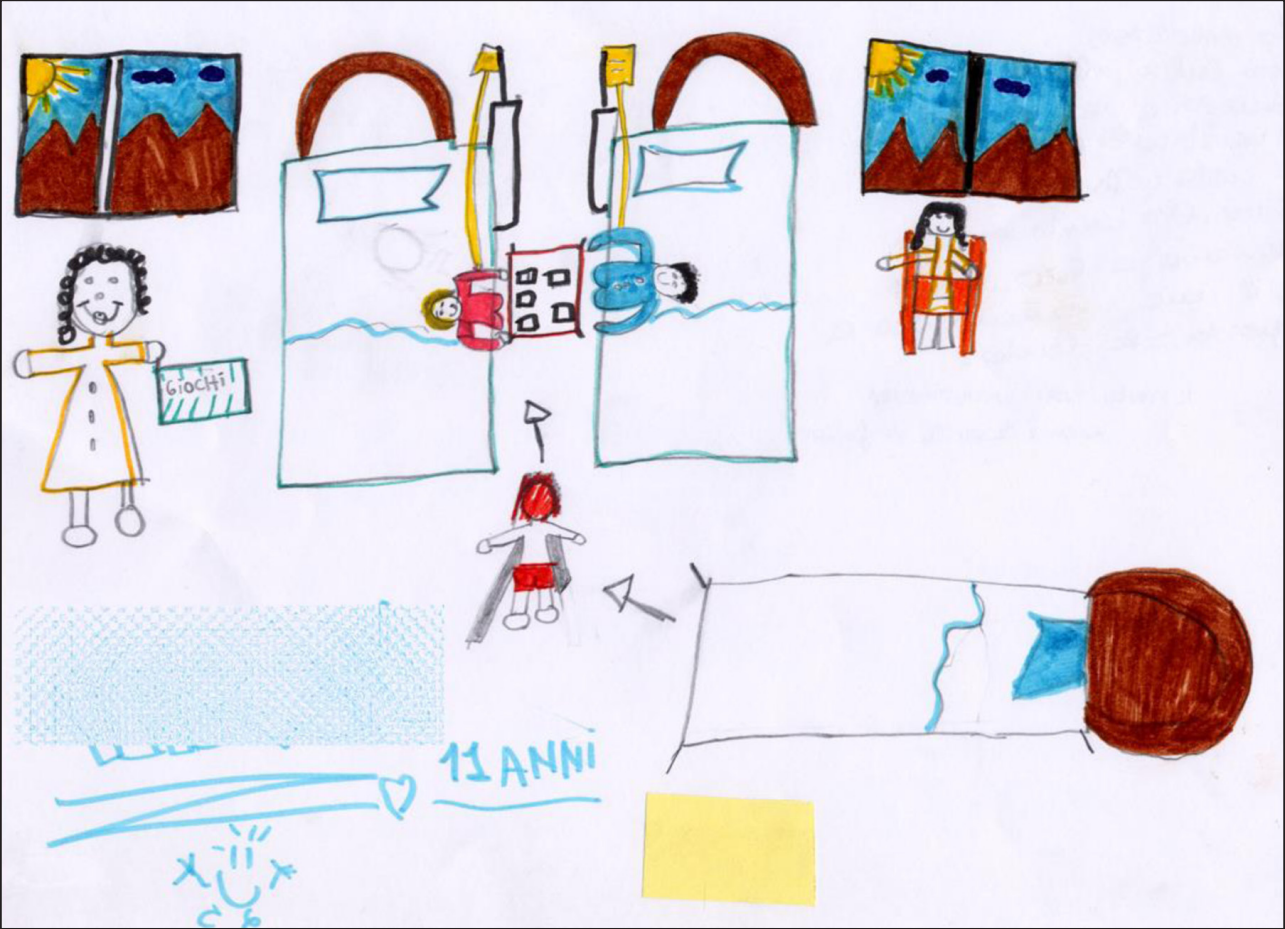
Karen, age 11. The doctor enters the room, and she sees two children playing cards and another child who is heading towards them. There is also a mother present, who is smiling at them.

Quite often, the presence of peers comes as a positive surprise and creates a relaxing and happy environment, as seen in Susan’s work (Figure 16), where the children occupy the center of the scene, while the beds are empty and are used as chairs, and the adults watch the children from a distance without interfering with them. It is as if the children in the picture need to be able to move and play within a separate space, which is safe because there is a group of adults watching over them from a distance.

### THE POWER OF PLAY

**Figure 17:**
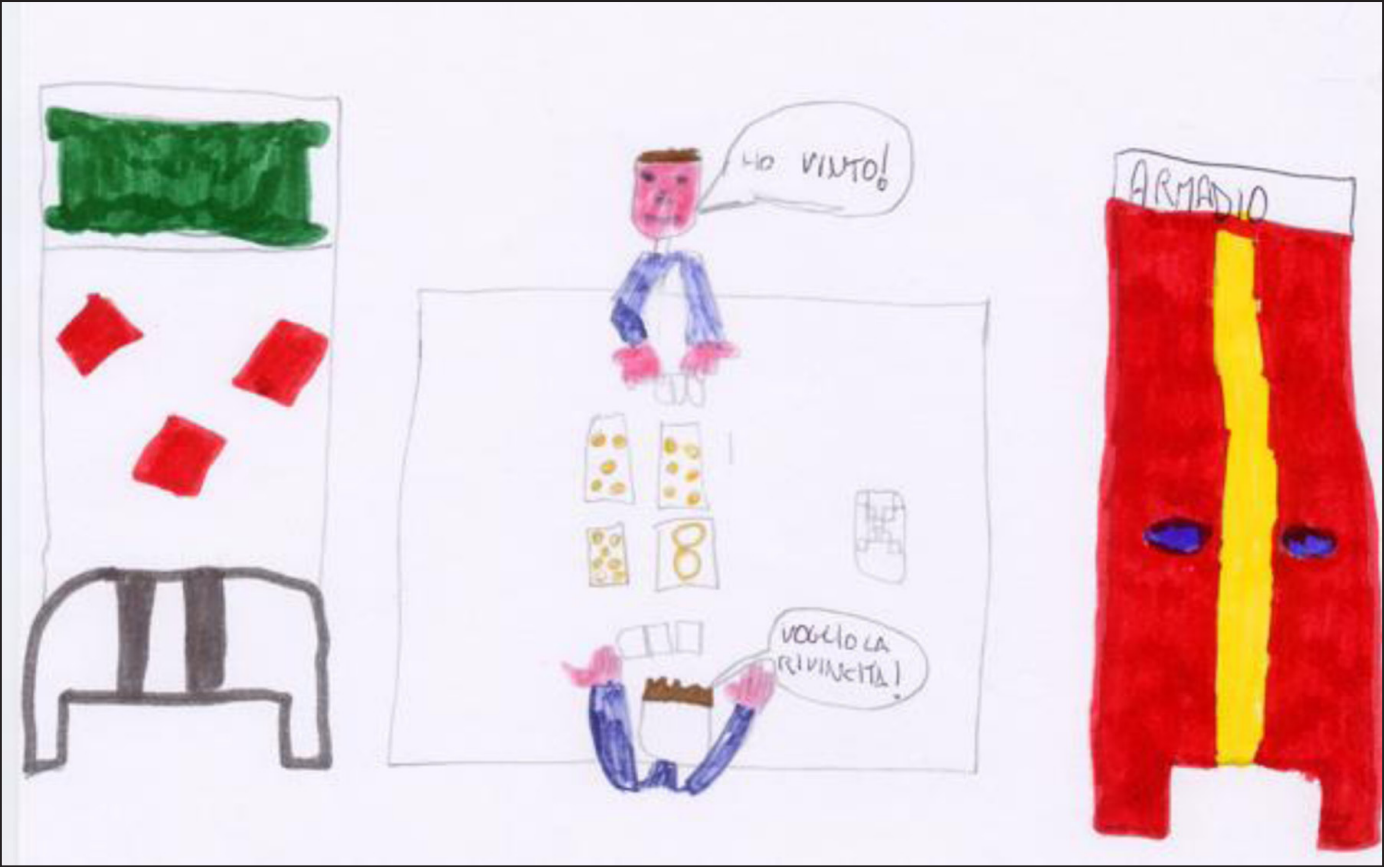
Marco, age 11. “Yesterday, when I was hospitalized, I realized there was another child in my room, on another bed next to mine. He is a boy, age 9, and his name is Pasquale. He is very friendly, and when I woke up this morning, he said, ‘Good morning to you,’ as if we were old friends. We soon became good pals, and this morning we played cards together. Pasquale lives in Naples, and when I leave the hospital, I won’t be able to meet him again. This makes me sad.”

Play is present in many of the children’s drawings and essays, and its presence appears to be linked with their ability to cope with the illness. Even more so than the hospital school, play appears to be a force capable of pushing the illness completely into the background. In Marco’s work (Figure 17), there is no trace of medical equipment. Instead, the center of the picture is occupied by a table with play activities. Children sit on each side and are interconnected by their activity, which acts as a mediator. The hospital bed (representing the illness) is empty; there is no more need for it.

**Figure 18:**
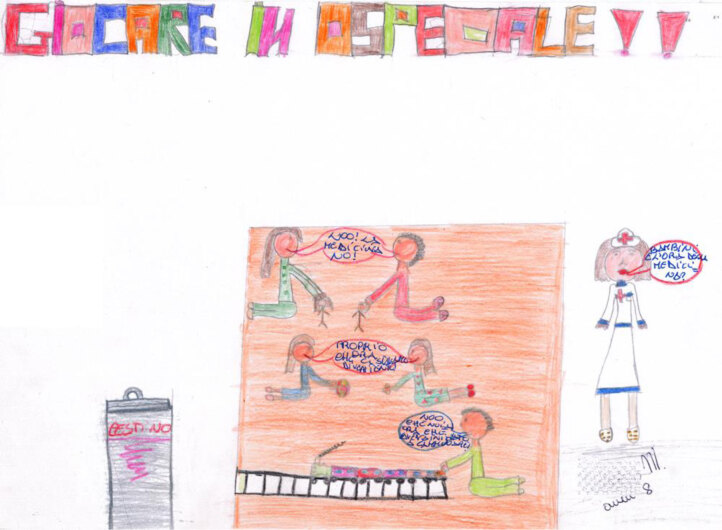
Carlo, age 8. The child depicts play as a safe area in the hospital setting.

**Figure 19:**
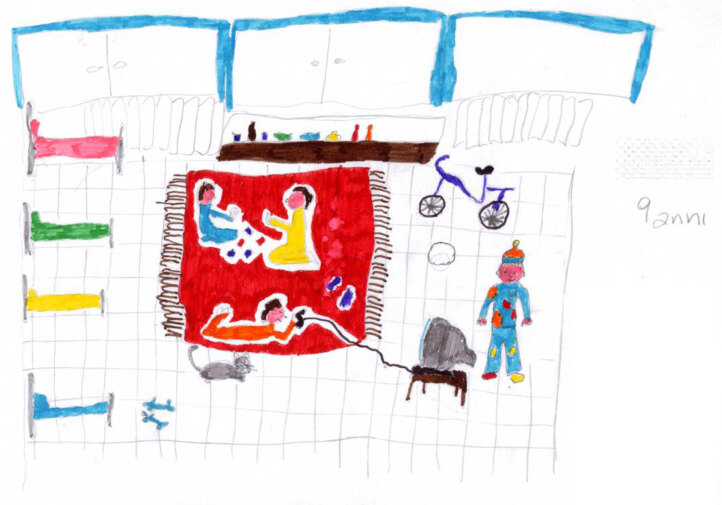
Pietro, age 9.

Play is also central in the works of Carlo (Figure 18) and Pietro (Figure 19), where a “safe area” is significantly represented by the orange or red carpet, which physically separates the healthy area where they are playing from the intimidating part of the rest of the hospital. The safe area is a shared space for children only, who are engaged in play activities. In Carlo’s work, a nurse comes into the room. She is confined to being outside of the shared area. Unfortunately, she has to get the children to take their medicine, but they protest because they have just started to have fun. The nurse looks like she is sorry to have to interrupt the children, indicating that she is sympathetic and understands them. However, the medicine must be taken. Nevertheless, the play area is large and crowded, and we can expect the child who has been called out to resume playing again soon.

**Figure 20:**
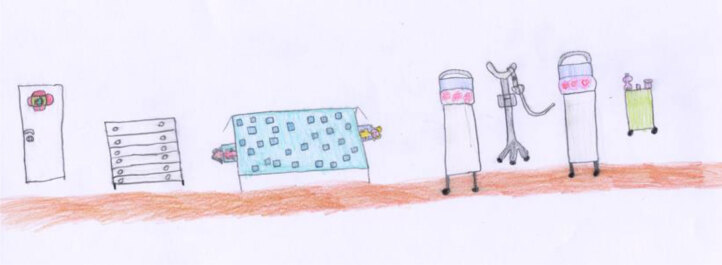
Maya, age 7. The child shows herself playing cards with a friend.

In Pietro’s picture (Figure 19), and also in Maya’s image (Figure 20), play takes up the central part of the scene, while the empty beds and the drip lines, which represent the illness, are pushed to the side as if they are part of a recent past event.

## DISCUSSION

All the narratives reported and commented on here reveal a great deal of complexity and heterogeneity in the way the children perceive, relay, and make sense of their life in the hospital and of their illness. Generally speaking, this is an indication of how children perceive illness and wellbeing as multidimensional constructs, largely related to their own agency and, therefore, to the instigative characteristic that may be present in the environment around them. In the eyes of the children, their illness is not linked only to their medical condition but is mediated by the opportunities to engage in chosen activities and participate in a supportive, everyday context, such as school.

### “ME AND MY ILLNESS”

Our first and second research questions investigated how children would explain and represent their illness and whether cognition is the only framework that we can use to analyze the children’s understanding of their illness.

In terms of the children’s understanding of their illness, our results are quite heterogeneous. Social and cognitive experiences of illness featured in the children’s narratives. As seen in the results section, several children’s works could be interpreted within Bibace and Walsh’s (1980, 1981) Piagetian framework. However, other works did not fit into this system, and some children seemed to rely on other ways of sense-making. These children present us with the need to adopt a socio-constructivist model to explain their understanding of their illness. As an example, we note Figures 3 and 4. Here, children who were in a hospital context that supports social and cognitive communication were probably able to develop an understanding of their illness through their zone of proximal development (Vygotskij, 1978). According to Vygotskij, the source of human cognition is based on the social and cultural word (Vygotskij, 1978). When working in a social context with someone more competent, children are capable of more complex and advanced actions.

We believe this is the case for Giovanni, who observed the work of his older roommate and, according to the local mediator, started asking his friend and the nurse about chemotherapy, blood cells, etc., and then drew his own picture, showing a level of understanding that he would not have reached alone. These findings, therefore, corroborate the theories speculating that some children make sense of their illness in a social-constructivist way (Bir & Podmore, 1990; McIntosh et al., 2012). On the contrary, when some kind of integration with the context is not possible, children’s images reflect a sense of isolation or broken parts, such as in the images depicted in Figures 1 and 13.

### THE IMPORTANCE OF RELATIONSHIPS AND COPING

The third research question was of a phenomenological nature, trying to identify what the most critical events, coping mechanisms, and relationships were for children while they were in the hospital.

Three major recurring helping systems were identified in the children’s narratives. The first was the presence of a primary caregiver and, in some cultures, also of other extended family members; the second was the chance to establish meaningful relationships with someone who was there to take care of the “healthy part” of the child, and not only to cure the disease. Finally, the third helping system was represented by play.

Their relationships with other people are the main means of dealing with hospitalization for most of the children. The children’s (and parents’) primary suffering stems not only from the illness per se, but from the threat to their sense of safety, and this type of suffering can be managed by maintaining good relationships with other people and continuing to perform normal childhood activities that bring pleasure to children’s everyday lives (Brazelton, 1992). For example, for older children, the presence of a roommate to play with is important, and in the child’s immediate environment, the medical and illness-related equipment tends to fade whenever he/she meets with other significant people. Many of the children’s drawings appear to be split into two sections (e.g., Figures 13, 19 and 20). On one side, there are elements such as medical equipment, manifestations of pain, and health practitioners that act as reminders of the illness or treatment; on the other side, there are the elements that recall a dynamic, and child-like life, such as positive self-images, other people with whom they have positive relationships, windows to the outside world, and play activities and toys.

The dichotomy in the children’s drawings between the illness and the healthy part of the self may be used to assess their perception of their agency and coping capabilities when faced with an illness. Thus, comparisons of the surface ratios of these two aspects could provide an insight into the child’s psychological state and help determine the appropriate social and educational activities or psychological therapies needed. This hypothesis warrants further investigation and corroboration in future studies.

Our fourth research question concerned the importance of play. Play is another vital factor that emerges from the children’s narratives, not only as a social means but also as a coping mechanism. The children’s narratives clearly indicate that recreational activities have great power over illness: They are capable of relegating disease and the medical equipment to the background and making it a secondary concern. It is as if play creates a “bridge over troubled water” (Capurso & Ragni, 2016), a safe space in children’s mind where they can go back to being themselves. They can use it to develop the healthy part of their personality, with a desire to learn, to grow old, and to make new friends. This finding confirms those of previous studies showing that play is used as a coping mechanism (Russ, 2004).

The children’s narratives tell us about their discovery of play and “good” people, often with a sense of surprise. Many children end their essays or poems saying: “I did not know that hospital life could also be good.” According to Piagetian and neo-Piagetian views (Kohlberg, 1971; Piaget, 1932), good and evil cannot co-exist in children’s minds, so they are surprised every time they discover that while the hospital is not always a pleasant place to stay, there are also good people and enjoyable activities. However, when a child tells us that inside the hospital there is also “a nice and friendly roommate” or that the “physical therapists, even if they support Inter (the football team from Milan), are really good,” they are actually discovering something much more important. They are envisioning the therapeutic function of the medical institution; they can make sense of their reality and understand that the bad aspects of the treatment are linked to a greater good and, therefore, they can foresee their chances of recovery.

### IMPLICATIONS FOR PRACTICE

Our research may provide professionals with insight into children’s views, opinions, and values. We believe that some practice recommendations can be derived from our findings.

#### Educators

Some children showed that they did not know about the presence of different services, activities, and peers inside the hospital. Therefore, creating a set of informative materials to give to children before, or soon after, hospitalization is a good way to reassure them and to give them positive thoughts and start planning for future days to come.

Children showed a great deal of resilience through their narratives. If they cannot find or access a playroom, they will move a table between their beds and start playing there. Their need to affirm their essence through play and social connections – friendship – is very strong and is present in all the positive accounts of the hospital. Educators should sustain this positive force in hospitalized children by recognizing their healthy side through the creation of activities that encourage play, social connection, learning, and talking with others. Hospitalized children with siblings or close friends and relatives will probably want to maintain their connections with these significant individuals as well, and proper means and activities to maintain these types of relationships should be provided. Educators could probably also act as advocates for children in the hospital setting and encourage, when needed, the development of policies for the creation of specific children’s spaces that are rich with toys and games, and the availability of common spaces where children can spend time with friends whenever possible.

Another important aspect that can be inferred from several of the children’s narratives is their need to communicate. All activities that encourage communication and create mediators and contact between people (e.g., a letterbox for exchanging suggestions, school activities where children can interview medical practitioners) within the hospital should be encouraged by education professionals.

#### Medical practitioners

The importance of recognizing the complexity of children’s emotions, thoughts, fears, and concerns should encourage all practitioners to listen to children. Quickly going into the child’s room or standing by the door, asking. “how are you today?” does not mean communication is taking place. The narratives reported here show how children draw and explain their experiences and expectations when they feel that someone is really there for them, someone who is interested in making a difference based on what they have to say. A good way to start a conversation with children would be to ask them to draw their illness, describe their treatment, or write down some questions they want answered the next time they meet with a medical practitioner.

Practitioners should also make an effort to make the illness-related experiences less significant in the child’s life. The drawings and narratives reported here show the deep need of the children to have an area where they can feel safe and secure. Those areas should be provided and respected. The same goes for time and schedules. Every effort should be made to delineate clearly what times of the day are for therapy and diagnostics and when the children get to have their own personal time.

### LIMITATIONS OF THE STUDY

Despite the importance of its findings, the current study is subject to a number of limitations.

First, as with other phenomenological studies, the narratives reported here are subjective and, therefore, not generalizable. Furthermore, the original sample from whom the narratives were taken and used in this study was self-selected. While this study reported on narratives collected from multiple sites, the sample remains limited to Italy. Given the fact that culture influences perception and sense-making of illness and disease, and considering that clinical realities are also culturally constructed (Kleinman, Eisenberg, & Good, 2006), the findings reported here cannot be generalized to different cultural settings.

Second, concerning the primary study (Bianchi di Castelbianco et al., 2017), we do not know if, and how many, children refused to take part in the proposed tasks, or whether the local mediator chose to exclude some children, and if so, why this was the case.

Further, given the exploratory nature of the study, and due to privacy issues, we did not consult the medical data associated with each child’s narrative. This consequently, led us to overlook one of the key components of IPA research, the idiographic details relating to the patient’s medical history.

Despite these limitations, this study provides meaningful insights into children’s hospital experiences. It also shows how children’s voices can be heard through the use of age-tailored narrative tools such as writings and drawings. The narratives reported here provide several suggestions for educators and medical practitioners who may wish to improve their work by understanding and listening to children in a hospital setting.

### IMPLICATIONS FOR FURTHER RESEARCH

The findings of the current study have opened up several areas that could be addressed in the future.

Future research would expand on our knowledge of how play and social connectedness support children’s abilities to cope with adversity during illness. While the current study adds to our understanding of the development, and environmental conditions that children perceive as helpful, further longitudinal research could investigate how patients’ narratives and pictorial representations change over the period of hospitalization, and cross-sectional studies could address the question of how children’s perceptions change across different age cohorts and cultural contexts. The ratio between illness-related objects and representations of healthy elements in pictorial representations could reflect the way that children with medical conditions perceive their sense of agency, and their coping strategies from a psychological standpoint, could be further investigated in a multimethod design where children’s drawings are connected to objective measurements of their anxiety and coping capabilities.

Finally, if future studies could associate individual medical data and children’s narrative of their illness, a better understanding of the interlink between the medical history, the child perception of the illness and the effect of the local culture could emerge.

## CONCLUSION

Thanks to the use of narrative modes of thought, this study shows how children were able to connect with a psychological reality that would otherwise have remained internalized because it was not possible to share it with anybody. Children represent themselves and their condition in a broad variety of ways, which is difficult to code and array using a single model. The use of IPA enabled us to organize these narratives and provide an overview, not only of the most manifest aspects but also of some of the more profound emotional and psychological aspects of their narratives.

Children with a medical condition actively construct meaning for their illness experience by combining what is inside and around them: their individual experience as patients, the organization of their social and cultural world, their feelings and those exhibited by their caregivers, and the way the medical system is organized and perceived. Children are not just playing or looking for friends to have fun with. They play and relate to others to act and feel alive. When they find a rich and stimulating environment, hospitalized children can make sense of their reality, and their present and future perspective becomes active and full of hope. On the contrary, when they find themselves in a social-emotional vacuum, their body image and sense of the future can be compromised.

Educators, teachers, psychologists, and practitioners from all multidisciplinary hospital teams can make a difference in the future of a child with a medical condition through creating a suitable environment and by establishing and empowering existing relationships. This can include taking note of the children’s narratives and opinions. For example, when re-organizing a ward or a children’s service, children’s voices should be “heard” through the use of their narratives, as is demonstrated by this study. Our study presents a simple way to improve the care of children in hospital by truly listening to them and ascertaining their needs, such as the requests from this concluding child’s work:

“Born and living here, I never had the chance to know the world outside. It looks so beautiful and lively. I hope to recover quickly, but my doctors don’t believe I will and, in the meantime, I wonder what it is like living in that world I can only watch it from my window. Last week I saw a tall and monstrous machine. I asked my mummy, ‘what shall we do?’ but she told me it was just a crane, which is used to build new houses. I was so scared! Yesterday I saw an airplane – this is what they call it – and it was flying so high in the sky. They told me it is so cool to go on a trip! I really would like to have a friend to play with and make jokes. We could have fun and explore the world together. It would be fantastic if my doctors would say I can finally recover and I can go to a real school with other kids. All I ask for is a bit of hope for the future. I wish some day I could walk into that world that right now, I can only observe from far away.” (Giuseppe, age 12)

## References

[1] Angell, C., Alexander, J., & Hunt, J. A. (2014). “Draw, write and tell”: A literature review and methodological development on the “draw and write” research method. Journal of Early Childhood Research, 13(1), 17–28. DOI: 10.1177/1476718X14538592

[2] Barakat, L. P., Pulgaron, E. R., & Daniel, L. C. (2009). Positive psychology in pediatric psychology. In R. G. Steele & M. C. Roberts (Eds.), Handbook of pediatric psychology, 4th ed. (pp. 763–773). New York, NY, US: The Guilford Press.

[3] Barlow, J. H., & Ellard, D. R. (2006). The psychosocial of children with chronic disease, their parents and siblings: An overview of the research evidence base. Child: Care, Health and Development, 32(1), 19–31. DOI: 10.1111/j.1365-2214.2006.00591.x 16398788

[4] Bianchi di Castelbianco, F., Capurso, M., & Di Renzo, M. (2017). Ti racconto il mio ospedale [Telling about my hospital]. Rome, Italy: Magi.

[5] Bibace, R., & Walsh, M. E. (1980). Development of children’s concepts of illness. Pediatrics, 66(6), 912–917. 7454481

[6] Bibace, R., & Walsh, M. E. (1981). Children’s conceptions of health, illness, and bodily functions. San Francisco, CA, US: Jossey-Bass.

[7] Bir, J. E., & Podmore, V. N. (1990). Children’s understanding of health and illness. Psychology & Health, 4(2), 175–185. DOI: 10.1080/08870449008408151

[8] Boztepe, H., Çınar, S., & Ay, A. (2017). School-age children’s perception of the hospital experience. Journal of Child Health Care, 21(2), 162–170. DOI: 10.1177/1367493517690454 29119813

[9] Brazelton, T. B. (1992). Touchpoints: Your child’s emotional and behavioral development. Boston, MA, US: Addison-Wesley.

[10] Bronfenbrenner, U. (2005). Making human beings human: Bioecological perspectives on human development. Thousand Oaks, CA, US: Sage Publications.

[11] Bruner, J. S. (1986). Actual minds, possible worlds. Cambridge, MA, US: Harvard University Press.

[12] Bruner, J. S. (1991). The narrative construction of reality. Critical Inquiry, 18(1), 1–21. DOI: 10.1086/448619

[13] Canevaro, A. (1976). I bambini che si perdono nel bosco: Identita e linguaggi nell’infanzia [Children who get lost in the woods: languages and identities of childhood]. Firenze, Italy: La nuova Italia.

[14] Capurso, M. (2015). Supporting children’s development through educational work: A bioecological perspective. Psychology and Education, 52(3–4), 34–48.

[15] Capurso, M. (2019). Communicating diagnoses and therapies to children [Comunicare ai bambini diagnosi e terapie]. Quaderni acp, 26(4), 174–178.

[16] Capurso, M., Lo Bianco, M., Cortis, E., & Rossetti, C. (2016). Constructing an explanation of illness with children: A sample case study of juvenile arthritis. Child Care in Practice, 22(3), 247–256. DOI: 10.1080/13575279.2015.1054788

[17] Capurso, M., & Ragni, B. (2016). Bridge over troubled water: Perspective connections between coping and play in children. Frontiers in Psychology, 7(1953). DOI: 10.3389/fpsyg.2016.01953 PMC5183588 28082926

[18] Child Stats. (2017). Number of children (in millions) ages 0–17 in the United States by age, 1950–2017 and projected 2018–2050. Retrieved from https://www.childstats.gov/americaschildren/tables/pop1.asp

[19] Compas, B. E., Jaser, S. S., Dunn, M. J., & Rodriguez, E. M. (2012). Coping with chronic illness in childhood and adolescence. Annual Review of Clinical Psychology, 8(1), 455–480. DOI: 10.1146/annurev-clinpsy-032511-143108 PMC3319320 22224836

[20] Coyne, I. (2006). Consultation with children in hospital: Children, parents’ and nurses’ perspectives. Journal of Clinical Nursing, 15(1), 61–71. DOI: 10.1111/j.1365-2702.2005.01247.x 16390525

[21] Dolto, F. (1985). La cause des enfants [The cause of children]. Paris, France: Laffont.

[22] Dudek, S. Z., Strobel, M. G., & Runco, M. A. (1993). Cumulative and proximal influences on the social environment and children’s creative potential. The Journal of Genetic Psychology, 154(4), 487–499. DOI: 10.1080/00221325.1993.9914747 8176391

[23] Einarsdottir, J., Dockett, S., & Perry, B. (2009). Making meaning: Children’s perspectives expressed through drawings. Early Child Development and Care, 179(2), 217–232. DOI: 10.1080/03004430802666999

[24] Fargas-Malet, M., McSherry, D., Larkin, E., & Robinson, C. (2010). Research with children: Methodological issues and innovative techniques. Journal of Early Childhood Research, 8(2), 175–192. DOI: 10.1177/1476718X09345412

[25] Goldhagen, J. (2003). Children’s rights and the United Nations Convention on the Rights of the Child. Pediatrics, 112(Supplement 3), 742–745. 12949339

[26] Grant, C. A. (1977). The mediator of culture: A teacher role revisited. Journal of Research and Development in Education, 11(1), 102–117.

[27] Griffiths, M., Schweitzer, R., & Yates, P. (2011). Childhood experiences of cancer: An interpretative phenomenological analysis approach. Journal of Pediatric Oncology Nursing, 28(2), 83–92. DOI: 10.1177/1043454210377902 20739585

[28] Harris, P. L. (1989). Children and emotion: The development of psychological understanding. Oxford, UK: Basil Blackwell.

[29] Harris, P. L., Olthof, T., & Terwogt, M. M. (1981). Children’s knowledge of emotion. Journal of Child Psychology and Psychiatry, 22(3), 247–261. DOI: 10.1111/j.1469-7610.1981.tb00550.x 7263789

[30] Jadue Roa, D. S., Whitebread, D., & Gareca Guzmán, B. (2018). Methodological issues in representing children’s perspectives in transition research. European Early Childhood Education Research Journal, 26(5), 760–779. DOI: 10.1080/1350293X.2018.1522764

[31] Jans, M. (2004). Children as citizens: Towards a contemporary notion of child participation. Childhood, 11(1), 27–44. DOI: 10.1177/0907568204040182

[32] Kleinman, A., Eisenberg, L., & Good, B. (2006). Culture, illness, and care: Clinical lessons from anthropologic and cross-cultural research. FOCUS, 4(1), 140–149. DOI: 10.1176/foc.4.1.140 626456

[33] Kohlberg, L. (1971). Stages of moral development. Moral Education, 1(51), 23–92. DOI: 10.3138/9781442656758-004

[34] Lansdown, G. (2011). Every child’s right to be heard: a resource guide on the UN committee on the rights of the child general comment no. 12. London, UK: Save the Children/United Nations Children’s Fund.

[35] Lansdown, G., Jimerson, S. R., & Shahroozi, R. (2014). Children’s rights and school psychology: Children’s right to participation. Journal of School Psychology, 52(1), 3–12. DOI: 10.1016/j.jsp.2013.12.006 24495491

[36] Lemerise, E. A., & Harper, B. D. (2014). Emotional competence and social relations. In K. Hansen Lagatutta (Ed.), Children and emotion. New insights into developmental affective science. Contributions to human development, 26, 57–66. Basel, Switzerland: Karger Publishers. DOI: 10.1159/000354353

[37] Leonhardt, C., Margraf-Stiksrud, J., Badners, L., Szerencsi, A., & Maier, R. F. (2014). Does the “Teddy Bear Hospital” enhance preschool children’s knowledge? A pilot study with a pre/post-case control design in Germany. Journal of Health Psychology, 19(10), 1250–1260. DOI: 10.1177/1359105313488975 23818510

[38] Malchiodi, C. A. (1998). Understanding children’s drawings. New York, NY, US: Guilford Publications.

[39] Martínez-Mejías, A., Úriz, M. S., Rivera-Pérez, C., & Garolera, M. (2017). Anxiety, hospital fears and conduct and behavioral alterations during pediatric hospitalization. Actas Espanolas de Psiquiatria, 46(2), 42–50. 29616712

[40] Masten, A. S. (2001). Ordinary magic: Resilience processes in development. American Psychologist, 56(3), 227. DOI: 10.1037/0003-066X.56.3.227 11315249

[41] McAdams, D. P., & McLean, K. C. (2013). Narrative identity. Current Directions in Psychological Science, 22(3), 233–238. DOI: 10.1177/0963721413475622

[42] McIntosh, C., Stephens, C., & Lyons, A. (2012). Young children’s meaning making about the causes of illness within the family context. Health, 17(1), 3–19. DOI: 10.1177/1363459312442421 22561292

[43] McIntosh, C., Stephens, C., & Lyons, A. (2013). Remember the bubbles hurt you when you cook in the pan: Young children’s views of illness causality. Psychology, Health and Medicine, 18(1), 21–29. DOI: 10.1080/13548506.2012.687829 22639797

[44] Obaid, K. B. (2015). Psychosocial impact of hospitalization on ill children in pediatric oncology wards. Journal of Nursing Health Sciences [on-line], 4(3), 72–78.

[45] Piaget, J. (1932). The moral judgment of the child. New York, NY, US: Taylor & Francis.

[46] Piaget, J. (1953). The origin of intelligence in the child. London, UK: Routledge & Paul.

[47] Quaye, A. A., Coyne, I., Söderbäck, M., & Hallström, I. K. (2019). Children’s active participation in decision-making processes during hospitalisation: An observational study. Journal of Clinical Nursing, 28(23–24), 4525–4537. DOI: 10.1111/jocn.15042 31430412 PMC7328781

[48] Roberts, M. C., Aylward, B. S., & Wu, Y. P. (2014). Clinical practice of pediatric psychology. New York, NY, US: Guilford Publications.

[49] Rushforth, H. (1999). Practitioner review: Communicating with hospitalised children: Review and pplication of research pertaining to children’s understanding of health and illness. The Journal of Child Psychology and Psychiatry and Allied Disciplines, 40(5), 683–691. DOI: 10.1111/1469-7610.00485 10433403

[50] Russ, S. W. (2004). Play in child development and psychotherapy: Toward empirically supported practice. Mahwah, NJ, US: Lawrence Erlbaum Associates Publishers. DOI: 10.4324/9781410609397

[51] Rutter, M. (1987). Psychosocial resilience and protective mechanisms. American Journal of Orthopsychiatry, 57(3), 316–331. DOI: 10.1111/j.1939-0025.1987.tb03541.x 3303954

[52] Sartain, S. A., Clarke, C. L., & Heyman, R. (2000). Hearing the voices of children with chronic illness. J Adv Nurs, 32(4), 913–921. DOI: 10.1046/j.1365-2648.2000.t01-1-01556.x 11095230

[53] Siegal, M., & Peterson, C. (2005). Children’s understanding of biology and health. Cambridge, UK: Cambridge University Press.

[54] Smith, J. A., & Dunworth, F. (2002). Qualitative methods in the study of development. In J. Valsiner & K. J. Connolly (Eds.), Handbook of developmental psychology (pp. 603–621). London, UK: Sage. DOI: 10.4135/9781848608306.n26

[55] Smith, J. A., Flowers, P., & Larkin, M. (2009). Interpretative phenomenological analysis: Theory, method and research. London, UK: Sage Publications.

[56] Smith, J. A., & Osborne, M. (2015). Interpretative phenomenological analysis. In J. A. Smith (Ed.), Qualitative psychology: A practical guide to research methods (3rd ed., pp. 25–52). London, UK: Sage Publications.

[57] United Nations. (1989). Convention on the Rights of the Child. In G. A. UN (Ed.), Treaty Series, 1577, 3. Geneva, Switzerland: Author.

[58] Vygotskij, L. S. (1978). Mind in society: The development of higher psychological processes (M. Cole Ed.). Cambridge, MA, US; London, UK: Harvard University Press.

[59] Walberg, H. J. (1969). Social environment as a mediator of classroom learning. Journal of Educational Psychology, 60(6 pt 1), 443. DOI: 10.1037/h0028499

[60] Ward, E., DeSantis, C., Robbins, A., Kohler, B., & Jemal, A. (2014). Childhood and adolescent cancer statistics, 2014. CA: A Cancer Journal for Clinicians, 64(2), 83–103. DOI: 10.3322/caac.21219 24488779

[61] West, A. M., Denzer, A. Q., Wildman, B. G., & Anhalt, K. (2013). Teacher perception of burden and willingness to accommodate children with chronic health conditions. Advances in School Mental Health Promotion, 6(1), 35–50. DOI: 10.1080/1754730X.2012.760920

[62] Witt, W. P., Weiss, A. J., & Elixhauser, A. (2014). Overview of hospital stays for children in the United States, 2012. Healthcare Cost and Utilization Project (HCUP), Statistical Brief, 187, 1–17. Retrieved from https://www.hcup-us.ahrq.gov/reports/statbriefs/sb187-Hospital-Stays-Children-2012.jsp

